# Machine-Learning-Assisted Aroma Profile Prediction in Five Different Quality Grades of Nongxiangxing Baijiu Fermented During Summer Using Sensory Evaluation Combined with GC×GC–TOF-MS

**DOI:** 10.3390/foods14101714

**Published:** 2025-05-12

**Authors:** Dongliang Shao, Wei Cheng, Chao Jiang, Tianquan Pan, Na Li, Mengmeng Li, Ruilong Li, Wei Lan, Xianfeng Du

**Affiliations:** 1 School of Food and Nutrition, Anhui Agricultural University, Hefei 230036, China; ahgkjc@163.com; 2 School of Biology and Food Engineering, Fuyang Normal University, Fuyang 236037, China; chengwei@fynu.edu.cn (W.C.); pantianquan@jzz.cn (T.P.); lina1036@jzz.cn (N.L.); limengmeng@fynu.edu.cn (M.L.); pennylrl@163.com (R.L.); 18956748848@163.com (W.L.); 3 Anhui Engineering Research Center for Functional Fruit Drink and Ecological Fermentation, Fuyang 236037, China; 4 Anhui WenWang Brewery Co., Ltd., Fuyang 236400, China; jiangchao_ww@hengshuilaobaigan.net

**Keywords:** Nongxiangxing baijiu (NXB), volatile organic compounds (VOCs), characteristic components, machine learning, discrimination and prediction of flavor

## Abstract

Flavor is one of the crucial factors that influences the quality and consumer acceptance of baijiu. In this study, we analyzed the volatile organic compound (VOC) profiles of five different quality grades of Nongxiangxing baijiu (NXB), fermented during the summer of 2024, using 2D gas chromatography time-of-flight mass spectrometry (GC×GC–TOF-MS). We employed machine-learning (ML)-based classification and prediction models to evaluate the flavor. For TW, the scores of the sensory evaluation for coordination and overall evaluation were the highest. TW contained the highest concentration of ethyl caproate; we detected 965 VOCs in total, including several pyrazine compounds with potential health benefits. Principal component analysis (PCA) combined with orthogonal partial least squares discriminant analysis (OPLS-DA) enabled us to distinguish different samples, with eight VOCs emerging as primary contributors to the aroma of the samples, possessing variable importance in projection (VIP) values > 1. Furthermore, we tested eight ML models; random forest (RF) demonstrated the best classification performance, effectively discriminating samples based on their VOC profiles. The key VOC contributors that showed quality-grade specificity included 1-butanol, 3-methyl-1-butanol, and ethyl 5-methylhexanoate. The results elucidate the flavor-based features of NXB and provide a valuable reference for discriminating and predicting baijiu flavors.

## 1. Introduction

Baijiu is a long-established alcoholic beverage that plays an integral role in social activities and the daily life of Chinese people [1]. The production process, including fermentation, distillation, and aging, is complex and variable [2,3]. The flavor is one of the critical factors that influences the quality and consumer acceptance of baijiu, as it exhibits distinct regional and brand characteristics [1,4,5]. During brewing, in addition to the effects of saccharification starters, raw materials, brewing fermentation containers, and brewing process, seasonal factors significantly impact quality [6,7]. Due to climatic influences, baijiu breweries often halt production in summer, resulting in ultra-long grain fermentation times [8]. Nongxiangxing baijiu (NXB) is one of the most popular flavor classes among the 12 categories of Chinese baijiu produced via natural solid-state fermentation [9]. The major NXB-producing regions in China include Sichuan Province and the Huanghuai area (including the Jianghuai region). NXB contains over 1300 different flavor compounds, contributing to its characteristic fragrant aroma, smooth texture, and lingering aftertaste [9]. Extended fermentation periods enhance its quality, primarily because of changes in volatile organic compounds (VOCs), which are influenced by variations in physicochemical indices and microbial community metabolism [8,10,11]. However, there is limited research on VOC-based differences between diverse quality grades of NXB fermented in summer and the relationships linking VOCs with sensory evaluations, particularly concerning major NXB-producing regions, such as the Jianghuai region.

For different samples, various pretreatment and chromatographic detection methods have been used to study VOC profiles [12]. At present, headspace solid-phase microextraction (HS-SPME) is regarded as a straightforward technique that eliminates the need for complex sample pretreatments, which has been applied to detect VOCs in alcoholic beverages [12,13,14]. Additionally, advanced analytical methods have been extensively used to investigate VOCs in baijiu [12]. For instance, 2D gas chromatography (GC×GC) can effectively separate multiple components from complex matrices, offering higher peak capacity, resolution, and sensitivity than their one-dimensional counterparts. When coupled with mass spectrometry (MS) of high-resolution, such as time-of-flight MS (TOF-MS), GC×GC technology has proven to be highly effective for separating and identifying aroma compounds in baijiu [12,15,16]. For example, using GC×GC-TOF-MS to detect VOCs in Guojing sauce-flavored baijiu resulted in the detection of nearly 1000 distinct compound peaks, among which 509 VOCs were identified [15].

Currently, machine learning (ML) is employed to build predictive models for aroma components and sensory characteristics by integrating multidimensional data such as GC-MS, gas chromatography-ion mobility spectrometry (GC-IMS), and sensory scores. This approach facilitates the quality assessment of baijiu and further aims to optimize fermentation and aging strategies [17,18]. Given the complex aroma composition of baijiu—which involves microbial metabolism, chemical reactions, and multiple variable interactions—traditional methods of data analysis make it difficult to fully explore the correlations between aroma substances and quality. In contrast, ML can analyze flavor composition more accurately and efficiently, predict quality, and perform sensory evaluations by leveraging multidimensional data (e.g., GC-flame ionization detection [FID], GC-MS, and sensory scores) in combination with automated feature selection and non-linear modeling [19,20].

In this study, we aimed to address the challenges in determining the quality grades of base NXB fermented during the summer. We evaluated differences in sensory analysis, identified the characteristics of VOCs using GC×GC–TOF-MS, and used ML-based classification and prediction models to assess the flavors. The results elucidate the flavor-based characteristics of NXB with different quality grades and provide a valuable reference for discriminating and predicting flavors of baijiu.

## 2. Materials and Methods

### 2.1. Samples and Reagents

#### 2.1.1. Collecting the Samples

We collected five base NXB samples with different quality grades from baijiu producers in Linquan County (32°87′ N, 115°19′ E) in Anhui Province, China. Baijiu grades were sampled from different stainless-steel storage tanks, which were used to store baijiu according to differences in grades, fermentation times, and workshops. The fermentation duration for the grains was approximately 180 days, from April to September 2024; during the summer, production was halted, and the standard fermentation period of approximately 90 days was exceeded. In late September 2024, after the distillation of fermented grains, the resulting base NXB was categorized into five distinct quality grades according to the workers’ production expertise and sensory evaluations. We designated these grades as TW, JT, YJ, YOJ, and WJ, respectively. The brewing process followed traditional NXB techniques with sorghum as the primary raw material, mid-temperature daqu as the saccharification agent, and mud pits as fermentation vessels [21].

#### 2.1.2. Chemicals and Reagents

The typical C7 to C30 alkanes (≥99.8%) were purchased from Sigma (St. Louis, MO, USA), chromatography-grade (≥98.5%) n-hexyl-d13 alcohol was acquired from C/D/N Isotopes (Pointe-Claire, QC, Canada), and analytical grade sodium chloride was obtained from Sinopharm Chemical Reagent Co., Ltd. (Shanghai, China). We sourced chromatography-grade anhydrous ethanol (≥99.8%) from Aladdin (Shanghai, China), and a Milli-Q system (Millipore, Billerica, MA, USA) was used to obtain ultrapure water.

### 2.2. Analysis of Sensory Flavor Characteristics

According to the national standard for NXB (the quality requirements for Baijiu, Part 1, Nongxiangxing Baijiu: GB/T 10781.1-2021 [22]), we evaluated sensory scores based on three aspects—“aroma”, “taste”, and “appearance”—with the samples being randomly numbered. Sensory analysis was performed according to the methods described previously [23], as well as the analytical technique for baijiu (GB/T 10345-2022) [24]. A panel of 15 trained assessors judged the samples. The sensory qualities of the samples were examined both quantitatively and descriptively. The intensity of aroma and taste were rated and recorded with a scale of 0 (not perceivable) to 5 (very strong), and those of intensity with increments of 0.5. We used the average value of each item as the final sensory score. In addition, the sensory evaluation and scores of the samples were assisted by members of the School of Biology and Food Engineering at Fuyang Normal University; with deep experience in assessing the quality of baijiu, we personally selected them through professional channels.

### 2.3. The Total Acids, Total Esters, and GC-FID Detection of Samples

The detection of total acids and esters in samples was according to the analytical method for baijiu: GB/T 10345-2022 [24]. The main flavor substances—ethyl caproate, ethyl acetate, and 1-propanol—were detected according to the same analytical technique [24].

### 2.4. Analysis of VOCs Using GC×GC–TOF-MS

#### 2.4.1. Preparing the Solution for the Internal Standard

The n-hexyl-d 13 alcohol, with an appropriate quantity, was accurately transferred to a 5 mL volumetric flask, dissolved to a concentration of 10 mg/L with a 50% (*v*/*v*) ethanol solution, and then stored at 4 °C in a refrigerator. A new solution was prepared before each experiment.

#### 2.4.2. The HS-SPME Method

The samples were extracted by HS-SPME according to the previously described approaches [25,26]. A saturated NaCl aqueous solution was used to dilute an appropriate amount of the sample to a 10% ethanol concentration (*v*/*v*) [27], and each 5 mL diluted sample was placed in a headspace vial (20-mL). Then, we added 10 µL of the internal standard solution (n-hexyl-d13 alcohol) and incubated the mixtures at 50 °C for 10 min. Extraction was performed using an HSPME fiber-coated with DVB/CAR/PDMS (50 µm × 30 µm × 1 cm; Supelco, Bellefonte, PA, USA). Prior to use, the SPME fibers were conditioned at 50 °C for 20 min.

After sample extraction, the SPME fiber was thermally desorbed with 250°C in the GC injection port for 5 min. Next, GC×GC–TOF-MS detection was performed according to the predefined parameters [28,29]. To ensure reproducibility, each sample was subjected to three replicate analyses. Aiming to prepare the SPME fiber for subsequent detection, the SPME fiber was conditioned in a heating chamber with 270 °C for 10 min following the injection process.

#### 2.4.3. The GC×GC–TOF-MS Method

The Agilent 8890A GC system (Agilent Technologies, Palo Alto, CA, USA) with a LECO Pegasus 4D instrument (LECO, St. Joseph, MI, USA) was applied, equipped with a split/splitless injector, dual-stage cryogenic modulator (LECO), and the detector (LECO) of TOF-MS. The GC×GC chromatographic separation was equipped with a column of DB-Heavy Wax (30 m × 250 μm I.D., 0.5 μm film thickness; Agilent) for the first dimension, while the second dimension was equipped with an column of Rxi-5Sil MS (2.0 m × 150 μm I.D., 0.15 μm film thickness; Restek, Bellefonte, PA, USA).

High-purity helium (>99.999%) was used as the carrier gas with a flow rate of 1.0 mL/minute for the GC×GC analysis. The temperature program was as follows: it was initially maintained at 40 °C for 3 min of oven temperature and increased to 200 °C with 6 °C/minute. Then, for the oven temperature, it was raised to 250 °C with 10 °C/minutes and maintained for 5 min. Meanwhile, the temperature of the second oven was 5 °C higher than that of the first oven. Additionally, the modulator temperature was maintained 15 °C above the temperature of the second column. For the GC injector, the temperature was set at 250 °C and implemented with a modulation period of 4 s.

For the TOF-MS analysis, a LECO Pegasus BT 4D system was applied to analyze different VOCs of the samples. The temperatures of the transfer line and TOF-MS ion source were both set to 250 °C. Moreover, using 200 spectra/second as the acquisition frequency, 70 eV with an *m*/*z* range of 35–550 was used for the mass spectrometer, operated in the electron impact ionization mode, and the detector voltage was set to 1960 V [28,29].

### 2.5. ML Model Construction

The most relevant features were identified based on the application of LASSO feature selections. For data pre-processing, we used the “StandardScaler” function to normalize the transformed features, which helps mitigate the risk of model overfitting [17,18]. We divided the dataset into training and test sets, with 20% of the data allocated to the test set using the train_test_split function. Further, aiming to balance the class distribution in the training set, we employed the synthetic minority oversampling technique (SMOTE), ensuring an adequate representation of minority classes for robust model training and prediction.

We used random forest (RF) and linear discriminant analyses as ML classifiers to categorize the VOCs of baijiu. We predicted the flavor score using ML regression models, such as linear regression and RF. Moreover, aiming to ensure balanced data during model training and prediction, SMOTE was applied to the training set [17,18].

### 2.6. Statistical Analysis and Mapping

We performed all measurements in triplicate, and we performed statistical analyses using SPSS (version 21.0; IBM, New York, NY, USA). Followed by Duncan’s multiple range test, we utilized a one-way analysis of variance (ANOVA) to assess the statistical significance of the data, and the significance level was set at *p* < 0.05.

#### 2.6.1. The Processing of GC×GC–TOF-MS Data

Using a Pegasus 4D workstation (LECO) for data collection and Chroma TOF software (4.50 version) for data analysis and processing, peaks with a signal-to-noise ratio exceeding 50 were automatically identified. We compared the peaks against the libraries of NIST 14 and Wiley 9 MS, selected the matching degree > 80% as the threshold, and further combined it with a retention time (RI) of C7–C30. We used MS to screen the peaks both with forward and reverse similarities > 700. We subsequently excluded compounds containing halogens and silicon elements [30].

To calculate the RI values of VOCs for C7–C30 n-alkanes, and then compare those with theoretical RI values, which we queried from the NIST online database (https://webbook.nist.gov/; accessed on 21 October 2024), we considered RI differences of 50 or less to be reliable [31]. The content of each VOC was calculated based on the internal standard, according to Equation (1).

(1) $$\mathrm{The}\;\mathrm{content}\;\mathrm{of}\;the\;VOC\;(µg/L)=\frac{\mathrm{Peak}\;\mathrm{area}\;\mathrm{of}\;\mathrm{compound}\;\times \;\mathrm{Internal}\;\mathrm{standard}\;\mathrm{amount}\;(µg/L)}{\mathrm{Peak}\;\mathrm{area}\;\mathrm{of}\;\mathrm{internal}\;\mathrm{standard}}$$

We used the relative odor activity values (ROAVs) to estimate the contribution of each VOC to the overall flavor and aroma. We generally regarded VOCs with ROAVs exceeding 1 to be pivotal flavor compounds; however, the VOCs with ROAVs < 1 were not regarded as the important aroma compounds of the sample [32]. We calculated the OAVs of each VOC, divided into an ROAV of 100 for the largest defined group, while correcting the ROAVs of other compounds, as shown in Equation (2).

(2) $$\mathrm{ROAV}=\frac{Peak\;B/TB}{Peak\;A/TA}$$

Peak A is representative of the maximum peak area of certain component, TA is representative of the odor value of the largest component, Peak B is representative of the peak area of the component to be measured, and TB is representative of the odor value of the compound to be measured [26].

#### 2.6.2. The Chemometric Analysis of GC×GC–TOF-MS Data

The GC×GC-TOF-MS data were mined based on the chemometric method; we filtered irrelevant and redundant data variables through pre-processing. We analyzed the variables semi-quantitatively according to the internal standard. The K-nearest neighbor algorithm was used to interpolate a small portion of missing values based on ML, which aims to simplify processing the multivariate and univariate analyses. Meanwhile, we removed variables with no statistical significance through a univariate analysis, screening potentially important VOCs preliminarily. We used version 22.0 of SPSS software (IBM, New York, NY, USA) to perform a single-factor ANOVA.

The false discovery rate (FDR) was applied to adjust the *p*-values, thereby minimizing false-positive results. Based on a significance level of *p* < 0.05 and a Pearson correlation coefficient of |r| > 0.6, we initially screened the variables. After scaling by unit variance, the data selected via a one-way ANOVA were subjected to multivariate statistical analyses. An unconstrained principal component analysis (PCA) was performed using version 14.1 of SIMCA software (Umetrics Academy, Umea, Sweden). We conducted a constrained orthogonal partial least-squares-discriminant analysis (OPLS-DA) to differentiate the VOCs by R software with the DiscriMiner package (version 6.3–73). Based on the BioDeep online analysis platform to generate cluster heatmaps of VOCs, we used Gephi (Version 0.9.1) to visualize the correlative network, while meeting the criteria of |ρ| > 0.6 and *p* < 0.05.

#### 2.6.3. Data Processing of ML Models

We used the Scikit-learn library to implement the ML models on the Jupyter Notebook (Python 3.8) platform, and the SHAP library was applied to calculate the samples’ SHAP values.

## 3. Results and Discussion

### 3.1. The Analysis of Sensory Flavor Differences and Characteristics Across Samples

**Figure 1:**
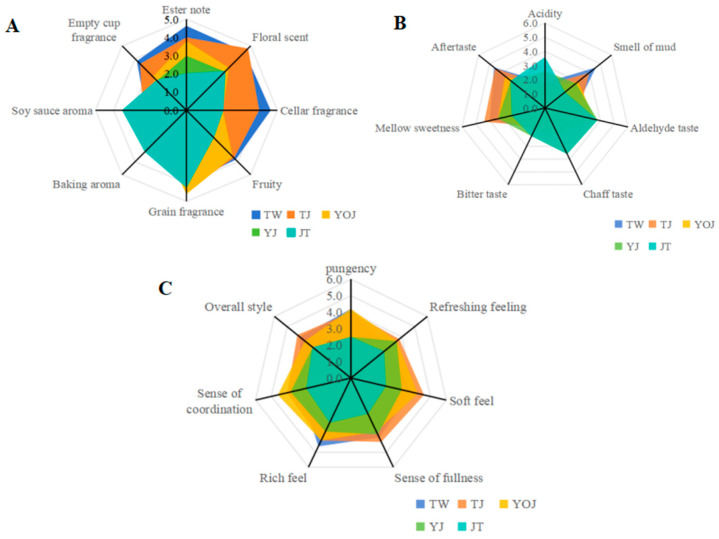
Sensory evaluation of samples designated as TW, JT, TJ, YJ, and YOJ: (**A**) aspects of “aroma”, (**B**) aspects of “taste”, and (**C**) aspects of “appearance”.

Past studies have characterized the sensory space to investigate the quality, complexity, typicality, and aging potential of alcoholic beverages (e.g., wine, baijiu), which are all regarded as sensory concepts [23,33,34]. As shown in Figure 1, the flavors of the five different quality grades of NXB were assessed according to the method of sensory evaluation, and we obtained a sensory-evaluation radar map.

The sensory flavor profiles of the five unique quality grades of NXB exhibited significant differences, with minimal variations observed between TW and TJ. However, substantial differences were noted in sensory properties when TW was compared with YJ or JT. The overall sensory characteristics of TW and TJ were described as having a prominent “ester note”, a “floral scent”, and a “fruity” and “cellar fragrance”, accompanied by a subtle “muddy taste” upon initial perception. In contrast, YJ was characterized by a more pronounced “pungency”, a “sense of fullness”, a “rich feel”, and a “sense of coordination”. Conversely, JT demonstrated a weaker “ester note”, “fruity” and “soy sauce aroma” attributes, “acidity”, an “aldehyde taste”, and a “chaff taste”. Additionally, JT lacked a “sense of coordination”, a “soft feel”, and had an overall unbalanced style.

In addition, the composite flavor profile of YJ was characterized by mildness, with a pronounced focus on grain “fragrance”, an “aldehyde taste”, and a “refreshing” sensation. It also featured appropriate levels of “acidity” and “pungency” but had less of a “cellar fragrance” and “muddy smell”. Overall, the flavor profiles of NXB at different quality grades were primarily determined by the comprehensive differences in the VOCs [10]. The TW and TJ samples demonstrated higher overall aroma scores, whereas the JT samples had lower taste scores, necessitating further in-depth research. Each baijiu sample possessed distinct flavor-based aspects, which we attribute to variations in the kinds and concentrations of trace flavor components. To accurately determine the concentration distribution of VOCs, it is essential to clearly understand the types of trace components present [8,35,36].

### 3.2. Detection and Differences of Total Acids, Total Esters, and the Main VOCs in the Samples

The taste of baijiu is influenced by the total ester, total acid, and main VOC contents, as shown for the five NXB samples in Table 1. The ethanol content of all samples exceeded 50% (*v*/*v*), with total acid levels greater than 1.73± 0.14 ^c^ g/L and total ester levels exceeding 8.35 ± 0.37 ^b^ g/L (Table 1). Maintaining a harmonious balance between sweetness, sourness, and bitterness determines the differences and aroma quality grades of baijiu, which are linked to the acceptability and preference of consumers [33]. The total acid content contributes to the acidity of baijiu. Organic acids—particularly acetic, hexanoic, butyric, and lactic acid—are the second most abundant compounds after esters. Moreover, the duration of the aftertaste is closely associated with certain organic acids with high boiling points [36]. As for total acid (Table 1), YJ exhibited the highest total acid value (3.47 ± 0.05 ^a^ g/L); however, JT had the lowest total acid content (1.73 ± 0.14 ^c^ g/L), indicating that YJ possessed an appropriately balanced “acidity”, whereas JT demonstrated an overall unbalanced style.

The total ester content of NXB varied significantly across the different quality grades (Table 1), with TW displaying the highest value (21.87 ± 0.11 ^b^) and YJ showing the lowest (8.35 ± 0.37 ^b^). Regarding ethyl caproate, TW demonstrated the highest concentration (1480.92 ± 0.05 ^a^), while JT had the lowest (89.14 ± 0 ^c^), suggesting that TW possessed a pronounced “pungency”. Regarding the ratio of ethyl caproate to ethyl lactate (Table 1), TW exhibited the lowest ratio (1480.92/120.37), whereas JT showed the highest ratio (89.14/129.09), implying that TW had a distinctive aroma profile characteristic of NXB. Ethyl lactate, ethyl butanoate, ethyl acetate, and ethyl hexanoate (which are present with high concentrations) are collectively referred to as the “four major esters in NXB”. Ethyl caproate serves as a key flavor component, whereas ethyl lactate acts as a flavor skeleton component. To enhance the aroma style of NXB, the concentration of ethyl caproate should be kept higher than that of ethyl lactate while maintaining an appropriate proportion [36].

### 3.3. Identification and Statistical Analysis of VOCs Based on GC×GC-TOF-MS

#### 3.3.1. Identifying the VOCs

**Figure 2:**
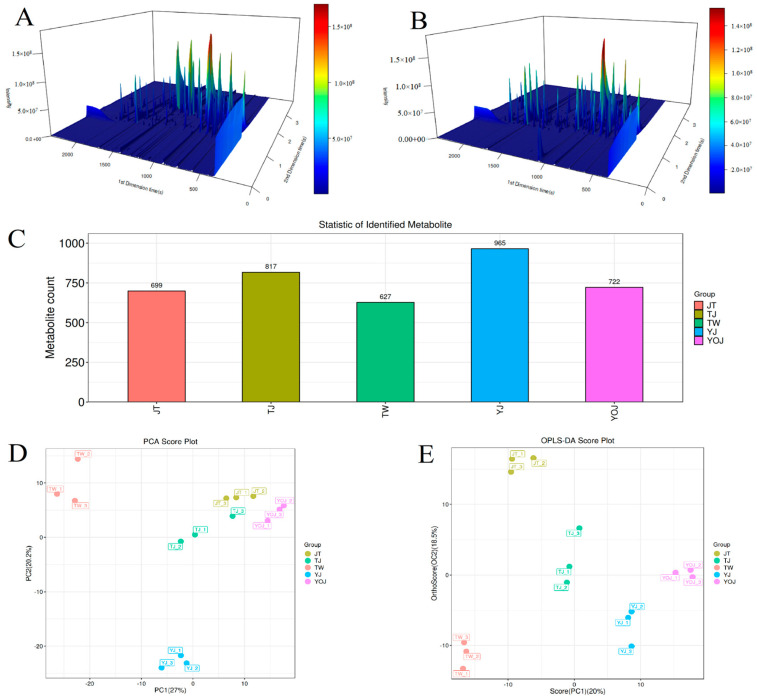
The VOCs identified in the five samples. (**A**) The total ion chromatogram (TIC) in 3D for TW. (**B**) The TIC in 3D for YOJ. (**C**) Quantitative comparison of VOCs. (**D**) PCA scores of VOCs. (**E**) OPLS-DA scores of VOCs.

Methods for detecting aroma components have advanced from traditional single-target analysis to multidimensional, high-sensitivity, and non-targeted approaches. The integration of high-resolution MS (such as GC×GC-TOF-MS) with chemometrics has significantly enhanced the accuracy of identifying aromatic substances [12,16]. The sensory characteristics of food are usually affected by most VOCs, whereas aroma plays an important role in shaping flavor characteristics and serves as a critical attribute affecting the satisfaction of consumers. For GC×GC-TOF-MS, compounds that are not fully separated based on the first dimension can be further resolved using two columns with orthogonal properties compared to GC-MS, thereby resulting in a broader range and higher quantity of VOC detection [12,15]. We achieved a clear and effective classification of VOCs in NXB using GC×GC-TOF-MS (Figure 2A,B). Additionally, the separation performance of VOCs in baijiu by GC×GC-TOF-MS is superior to that obtained by conventional GC-MS [23,36].

Table 2 lists the quantities of VOCs detected in the NXB using GC×GC-TOF-MS. In total, we identified 965 VOCs in YJ (Figure 2C), including 190 esters, 60 hydrocarbons, 84 alcohols, 49 ketones, 32 ethers, 40 carboxylic acids, 21 heterocyclic compounds, and 679 other compounds (Table 2). Among these, esters (19.69%) and alcohols (8.70%) were the predominant components. In contrast, we identified 627 compounds in TW (Figure 2C), including 149 esters, 37 hydrocarbons, 50 alcohols, 32 ketones, 25 ethers, 28 carboxylic acids, 25 heterocyclic compounds, and 281 other compounds (Table 2). Esters (23.76%) and alcohols (7.98%) were the primary constituents. In baijiu, most esters impart fruity and floral notes, while alcohols contribute to a sweet and smooth taste [34]. The aroma profile of NXB is a complex mixture of numerous compounds, each contributing to distinct features of different quality grades [30,36]. In this study, the results of VOC detection revealed that the flavor of NXB may be characterized by the combined roles of esters, alcohols, hydrocarbons, ketones, ethers, carboxylic acids, heterocyclic compounds, and other compounds.

Esters, which contribute distinctive aromas to baijiu [3], were the predominant VOCs detected in the samples. The ethyl acetate, ethyl caproate, ethyl lactate, and ethyl butyrate were the most abundant compounds (Table 1). The main furanones identified were 3-phenylfuran and 2-acetyl-5-methylfuran; we also detected 12 types of aldehydes, including decanal and heptanal. The main sulfur-containing compounds were 3-(methylthio)propanoic acid, methyl thiolacetate, ethyl ester, and thioacetic acid. Sulfur-containing compounds represent an essential class of odorants in liquors, with low concentrations, aroma thresholds, and unique aromatic properties. Both volatile and non-volatile sulfur-containing compounds play significant roles in determining the flavor profiles and quality of fermented beverages [37].

Further, we identified ketones, organic acids, and their derivatives, including 2-decanone, methyl isobutyl ketone, and methanesulfonic acid. Notably, we also detected pyrazine compounds such as tetramethylpyrazine, 2,3-dimethyl-5-ethylpyrazine, and 2,3,5-trimethyl-6-ethylpyrazine. Pyrazines are crucial aroma components with bioactive compounds in baijiu. Specifically, tetramethylpyrazine, a pharmacologically active compound, contributes a nutty and roasted flavor to baijiu while enhancing its health benefits, thereby significantly influencing the overall aroma and quality of baijiu [38].

VOCs with low sensory thresholds, combined with the other kinds of low-abundance VOCs, may further contribute to a distinctive flavor of food [26,36]. Certain micro-VOCs may be the primary contributors to the flavor and smell of baijiu. In sum, the trace contents of VOCs define the characteristic and dominant sensory evaluations of NXB, and the differences of quantity and content of these trace compounds largely determine the evaluation of their quality grades.

#### 3.3.2. The Comparison of Relative Amounts of VOCs

Esters primarily contribute “fruit, flower, sweet, and milk flavors”, imparting distinctive flavor profiles to baijiu. This is attributed to the diversity and concentration of esters, with 284 different esters identified in NXB [3]. Table 3 illustrates the relative contents of the VOCs across the five quality grades of NXB, indicating significant variations in ester content across the different samples. Specifically, the sample of TW exhibits the highest relative content of ester (60.73 ± 5.21 ^c^ µg/L), while YOJ has the lowest (49.00 ± 0.1 ^a^ µg/L). These findings align with the sensory evaluation outcomes presented in Figure 1, where TW is characterized by its prominent “floral scent” and “fruity aroma”. Overall, NXB contains a diverse range and high concentrations of ethanol esters, thereby corroborating previous findings [36].

YOJ has the highest relative concentration of alcohol (26.75 ± 1.77 ^b^ µg/L), while YJ has the lowest concentration (15.65 ± 1.98 ^bc^ µg/L). Alcohols are the key aromatic components of alcoholic beverages and significantly influence odor intensity. However, certain alcohols may impart unpleasant odors depending on their species and concentrations [39]. We detected the VOCs furan, ketones, aldehydes, and aromatic compounds in all five NXB samples, albeit at low levels. Despite their minor presence, these VOCs likely play the roles of complementing, coordinating, and modifying the sensory profile of baijiu [26,36].

#### 3.3.3. The Principal Component Analysis (PCA) and Partial Least-Squares-Discriminant Analysis (OPLS-DA) of the VOCs

Unconstrained PCA determines the weight of vital components based on the correlations of comprehensive indicators by reducing dimensionality [11,26]. The cumulative contribution rates of PC1 and PC2 are 47.2% (Figure 2D), partially describing the differences of VOCs in different samples. The five different quality grades of NXB are clearly separated without significant overlap, suggesting that the samples could be distinguished clearly. The VOC profiles differ significantly among different samples, as the sample points are all located in distinct quadrants and regions. Additionally, for TW and YJ, sample points positioned in separate quadrants are fairly distant from the other three sample points, implying that they exhibit greater dissimilarities than the other three samples. For JT, TJ, and YOJ, the sample points are clustered in nearly the same quadrant, suggesting that the VOC profiles of these three samples share many similarities.

In addition, we performed an unconstrained PCA and a constrained OPLS-DA to characterize the similarities and differences of VOCs among different samples. As seen in Figure 2E, NXB samples are distributed in distinct quadrants. The somewhat large distances between the sample points imply significant differences in their VOC compositions. In sum, the differences in the VOC profiles are pronounced, and both PCA and OPLS-DA effectively distinguish different NXB samples with different quality grades based on their VOC characteristics.

### 3.4. The Comparison of Key Differential VOCs and Their ROAVs in the Samples

#### 3.4.1. The Comparison of Quantities and Relative Contents of Key Differential VOCs

**Figure 3:**
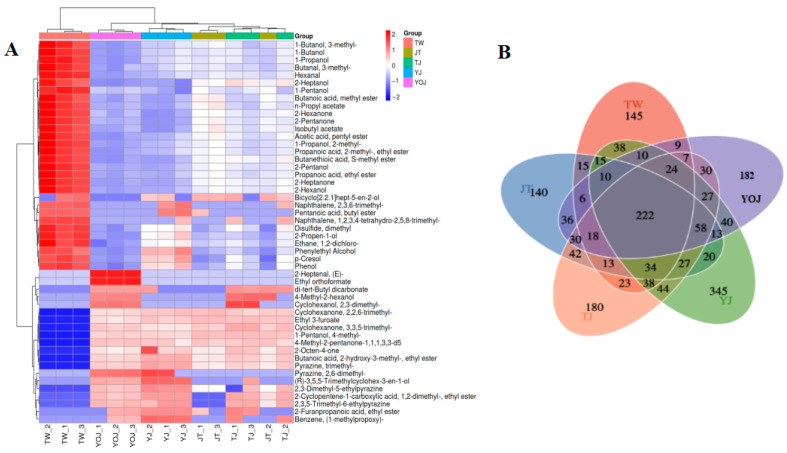
The cluster heat map of key differential VOCs (**A**) and Venn diagram with numbers indicating group material overlap (**B**) in the five different quality grades of the NXB samples. Adjusted to *p* < 0.05 (Tukey’s test).

The relative abundances of VOCs in the five NXB samples are visualized using a color-coded heat map (Figure 3). The individual samples are represented in the inner columns, specific metabolites are corresponded in the rows, and the dendrogram on the left portrays the hierarchical clustering with different VOC species. In the TW group, the relative contents of 1-butanol, 1-propanol, 1-butanol-3-methyl-acetate, 3-methyl-1-butanol, 1-hexanol, and an additional 24 VOCs are significantly elevated. Moderate concentrations of alcohol generally contribute to fruity and sweet aromas in baijiu; however, excessive alcohol content may impair its aroma and flavor profile, potentially introducing a pronounced bitter taste [36].

We identified 222 VOCs across the different samples, and the total number of differential VOCs varied among the samples (Figure 3B). These differential VOCs may serve as critical factors contributing to the sensory flavor differences between samples [17,18]. Consequently, further research on the quantity and composition of differential VOCs is necessary to explicitly define the quality grades of NXB.

#### 3.4.2. The Comparison of ROAVs of Key Differential VOCs in the Samples

**Figure 4:**
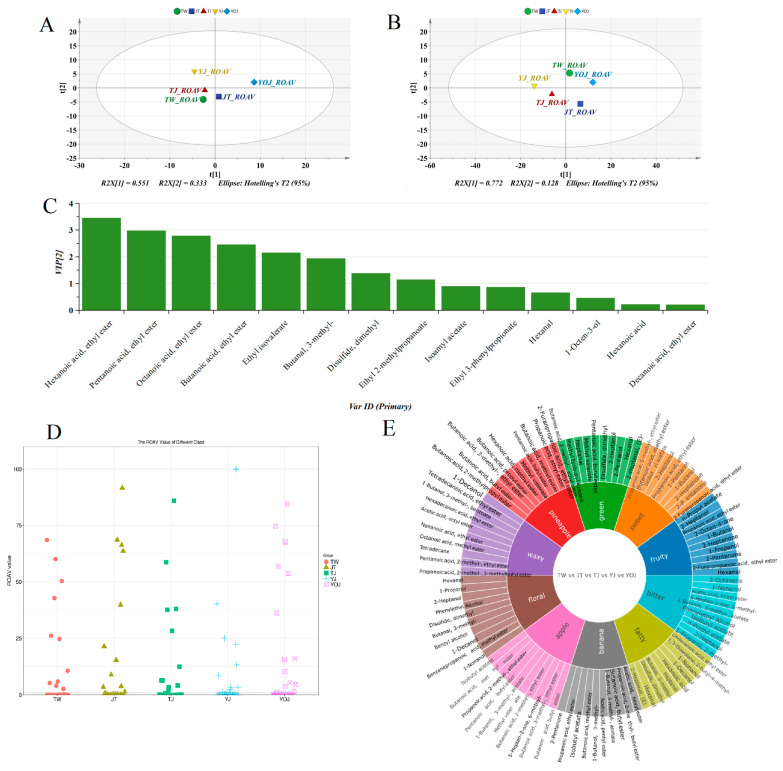
Coordinate VIP analyses based on the ROAVs of key differential VOCs in the samples. (**A**) PCA. (**B**) OPLS-DA. (**C**) VIP value. (**D**) Scatter plot of ROAVs. (**E**) Aroma contribution chart of key differential VOCs.

For the key differential VOCs of the samples (Table S1), the cumulative interpretation rates of PC1 and PC2 were 55.1% and 33.3% in the PCA (Figure 4A), highlighting distinct variations in key differential VOCs among different samples. In addition, the results of OPLS-DA (Figure 4B) for the key differential VOCs aligned well with the findings of PCA, confirming the differences in the NXB samples across various quality grades. When identifying key differential VOCs, the variable importance in projection (VIP) values reflect the relative importance of the variables in the model of OPLS-DA, and a higher VIP value indicates a greater contribution to the model [26]. We calculated the VIP values based on the ROAVs by OPLS-DA (Figure 4C), which were based on the dates of the key differential VOCs in the samples (Table S1). Eight VOCs (hexanoic acid ethyl ester, pentanoic acid ethyl ester, octanoic acid ethyl ester, butanoic acid ethyl ester, butanoic acid-3-methyl-ethyl ester, 3-methyl-butanal, dimethyl-disulfide, and propanoic acid-2-methyl-ethyl ester) exhibited VIP values exceeding 1, underscoring their critical role as distinguishing components among the samples.

Overall, the VOCs differences were statistically significant among samples, which served as the key indicators of VOCs differences in NXB across five different quality grades. As shown in Figure 4D, most of the key differential VOCs have ROAV values that do not exceed 1. Additionally, we identified 11 key differential VOCs in the five samples with ROAV values exceeding 1, including eight esters (butanoic acid-ethyl ester, octanoic acid-ethyl ester, butanoic acid-3-methyl-, ethyl ester, hexanoic acid-ethyl ester, 1-butanol-3-methyl-acetate, benzenepropanoic acid-ethyl ester, pentanoic acid-ethyl ester, and propanoic acid-2-methyl-ethyl ester) and three other VOCs (hexanal, dimethyl disulfide, and 3-methyl-butanal). These VOCs play important roles in the shaping of aroma characteristics of different NXB samples (Figure 4E, Table S1). Furthermore, Figure 4E illustrates that the key differential VOCs contributing to floral aroma include hexanal, 1-propanol, 2-heptanol, phenylethyl alcohol, dimethyl disulfide, 3-methyl-butanal, benzyl alcohol, 1-decanol, 1-nonanol, and benzenepropanoic acid-methyl ester.

#### 3.4.3. The Network Diagram of Relationships Between Various VOCs for Imparting the Unique Sensory of Aroma Characteristics Among Samples

**Figure 5:**
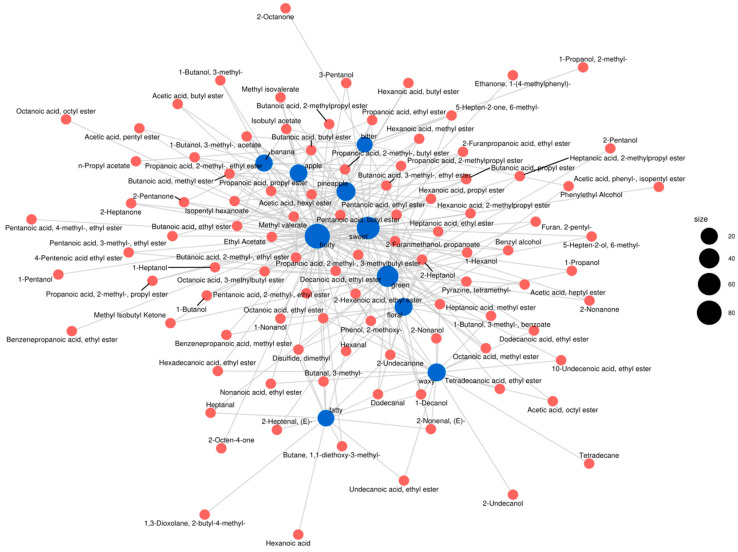
The network of relationships between the sensory flavor characteristics and flavor substances in the samples. Note: a blue circle represents a sensory feature, a red circle represents a flavor compound. The larger the blue circle, indicating the more types of flavor compound associated with that sensory feature, and the more important of this sensory feature.

Using Flavor DB2 and iGraph tools, the network diagram was built to visualize the relationships among different flavor compounds that contribute to the unique sensory of aroma characteristics [26]. By default, we selected the top ten sensory features for the network diagram. Notably, the sensory features of fruity, sweet, and pineapple were associated with more than 10 flavor compounds (Figure 5). For instance, fruit-aroma-related substances (which constitute the primary flavor components of NXB) are linked to compounds such as 3-methyl-1-butanol acetate, 1-butanol, 3-methyl-1-butanol, 1-hexanol, 1-propanol, and 66 additional flavor substances.

In addition, tetramethylpyrazine was associated with green, floral, and sweet sensory characteristics [26]. Tetramethylpyrazine, a distinctive flavor substance, typically imparts baijiu with a nutty and baked flavor. This compound can be generated through the Maillard reaction, microbial synthesis, and microbial metabolism during the solid-state fermentation of baijiu, which occurs while brewing NXB [33,36]. Few studies have comprehensively investigated the correlations among sensory evaluations, key differential VOCs, prolonged fermentation processes, and different quality grades. Furthermore, since VOCs significantly influence sensory attributes, employing GC-MS in conjunction with an electronic tongue and an osmometer, combined with sensory evaluations, is critical for assessing the NXB with different quality grades fermented over an extended period.

### 3.5. Development of the Classification and Prediction Models for Baijiu Flavor Based on ML Algorithms

To address the challenges of omics technologies, ML can uncover functionally important biological relationships and mechanisms from complex, multidimensional omics data through algorithm optimization and iterative model training, demonstrating a great potential for the processing and analysis of data [40]. This study performed a comprehensive and systematic analysis of VOCs in different quality grades of NXB samples, which revealed substantial variations in flavor-related chemical profiles across the different quality grades.

**Figure 6:**
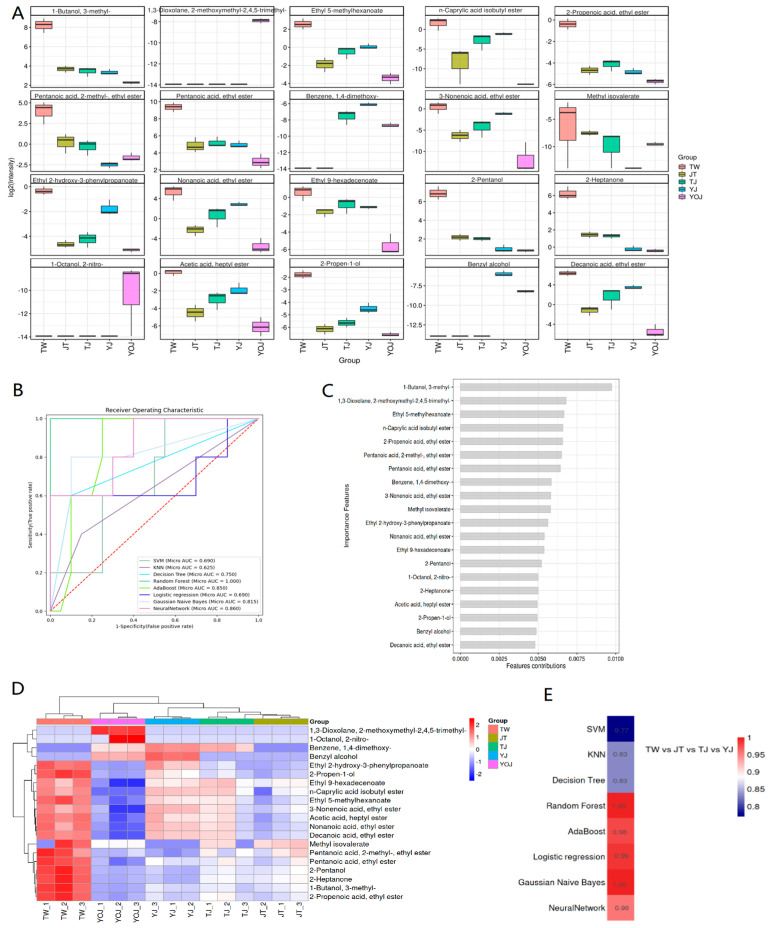
Box diagram of characteristics of VOCs (**A**), ROC diagram (**B**), ranking of the characteristic importance of the VOCs (**C**), heat map showing the characteristic importance of the VOCs (**D**), and AUC heat maps (**E**) based on ML.

A quantitative analysis of 20 representative VOCs enabled us to identify distinct distribution patterns (Figure 6A,C) with specific compounds, such as 3-Methyl-1-butanol, ethyl 5-methylhexanoate, 1,3-dioxolane, and 2-methoxymethyl-2,4,5-trimethyl, which demonstrated strong quality grade specificity (Table S2). To assess the discriminatory power of these compounds, we applied multiple ML models, including RF, neural networks, and Gaussian-naïve Bayes models. Among these models, the RF model achieved the highest performance (area under the curve [AUC] = 1.000) (Figure 6B,E), and the feature importance analysis confirmed that the aforementioned compounds significantly contributed to classification accuracy. Furthermore, the hierarchical clustering of VOC abundance highlighted systematic differences among the sample groups (Figure 6D), emphasizing the unique expression patterns for several key compounds in YOJ; this supports the existence of quality-grade-specific chemical signatures. Collectively, these findings indicate that VOCs serve as critical flavor biomarkers for distinguishing NXBs of different quality grades, and that ML models provide a reliable computational framework for quality assessment in baijiu production.

## 4. Conclusions

The complex flavor of NXB stems from the interaction of hundreds of VOCs and other non-volatile compounds at the physicochemical and sensory levels, which makes it challenging to accurately perceive NXB. In this study, we analyzed five representative NXB samples with different quality grades by combining sensory evaluation, GC×GC-TOF-MS detection, and classification and prediction models of ML. The sensory scores for the coordination and overall evaluation of TW were the highest among the descriptors of NXB with different quality grades evaluated. The content of ethyl caproate in TW was the highest, and we detected a total of 965 VOCs, including several kinds of pyrazine compounds, along with health benefits. The five samples could be distinguished based on PCA and OPLS-DA, while eight VOCs with VIP values > 1 were the main flavor components. In this study, we characterized different substances and their flavor associations in different quality grades of NXB, thus providing a reference for predicting aroma characteristics. Future studies should further optimize the ML model, expand the sample range, and combine multiple omics data (such as metabolomics) to analyze the synthesis pathways of different flavor substances of baijiu and provide theoretical support for detecting and improving baijiu product quality.

## Figures and Tables

**Table 1:** The detection results of the main physicochemical indices in different samples (ethanol, total acids, total esters, and the main VOCs detected by GC-FID).

Category	CAS	Unit	Samples				
			TW	TJ	YOJ	YJ	JT
Ethanol	/	*v*/*v*	70.45 ± 0.08	67.75 ± 0.17	64.8 ± 0.26	53.46 ± 0.33	68.53 ± 0.06
Total acid	/	g/L	1.89 ± 0.33 ^a^	2.26 ± 0.65 ^a^	2.41 ± 0.05 ^b^	3.47 ± 0.05 ^a^	1.73 ± 0.14 ^c^
Total ester	/	g/L	21.87 ± 0.11 ^b^	12.78 ± 0.1 ^a^	8.78 ± 0.23 ^a^	8.35 ± 0.37 ^b^	19.51 ± 0.05 ^d^
Acetaldehyde	75-07-0	mg/100 mL	18.8 ± 0.75	11.00 ± 0.05 ^a^	11.06 ± 0 ^c^	9.42 ± 0.15 ^a^	18.53 ± 0.23 ^a^
Methanol	67-56-1		8.46 ± 0.23 ^a^	10.66 ± 0.15 ^a^	16.00 ± 0.1 ^a^	21.97 ± 0.65 ^a^	8.39 ± 0.04 ^a^
Ethyl acetate	141-78-6		913.00 ± 0.1 ^a^	391.68 ± 0.05 ^b^	400.57 ± 0.05 ^b^	111.18 ± 0.17 ^a^	1031.19 ± 0.65 ^a^
n-propyl alcohol	71-23-8		83.08 ± 0.13 ^a^	73.69 ± 0 ^c^	7141 ± 0 ^b^	55.54 ± 0.05 ^a^	87.27 ± 0.03 ^b^
Sec-butyl alcohol	78-92-2		23.34 ± 0.23 ^a^	13.93 ± 0.05 ^d^	13.49 ± 0.17 ^a^	6.06 ± 0.53 ^a^	23.00 ± 0.1 ^a^
Acetaldehyde diethyl acetal	105-57-7		29.36 ± 0.61 ^a^	14.82 ± 0.15 ^a^	12.96 ± 0.05 ^b^	6.24 ± 0.15 ^a^	26.73 ± 0.17 ^a^
Isobutanol	78-83-1		15.59 ± 0.15 ^a^	11.44 ± 0.03 ^b^	10.65 ± 0.15 ^a^	6.17 ± 0.23 ^a^	17.35 ± 0.05 ^d^
n-butanol	71-36-3		84.24 ± 0.23 ^a^	74.00 ± 0.1 ^a^	66.76 ± 0.05 ^b^	48.83 ± 0.25 ^a^	81.96 ± 0.23 ^a^
Ethyl butyrate	105-54-4		160.53 ± 0 ^b^	78.07 ± 0.22 ^a^	76.79 ± 0.05 ^a^	28.65 ± 0.25 ^a^	177.00 ± 0.1 ^a^
Iso-amyl alcohol	123-51-3		34.38 ± 0.18 ^a^	28.59 ± 0.23 ^a^	21.83 ± 0.05 ^b^	20.45 ± 0.15 ^a^	31.08 ± 0 ^b^
Ethyl lactate	97-64-3		120.37 ± 0.05 ^b^	179.75 ± 0.13 ^d^	247.24 ± 0 ^c^	523.08 ± 0.25 ^d^	129.09 ± 0.05 ^b^
Ethyl caproate	123-66-0		1480.92 ± 0.05 ^a^	987.32 ± 0.23 ^a^	510.91 ± 0.15 ^a^	312.05 ± 0.23 ^a^	89.14 ± 0 ^c^

Note: Means ± standard deviations (n = 3) followed by different letters in each row indicate the significant differences based on the Duncan’s test (*p* < 0.05).

**Table 2:** Quantification of VOCs detected in the NXB samples by GC×GC–TOF-MS.

Group	Esters	Hydrocarbons	Alcohols	Ketones	Ethers	Carboxylic Acids	Heterocyclic Compounds	Others	Total
TW	149	37	50	32	25	28	25	281	627
JT	141	56	69	42	22	33	17	319	699
TJ	161	70	68	40	23	38	23	555	817
YJ	190	60	84	49	32	40	21	679	965
YOJ	144	33	78	42	27	33	20	345	722

**Table 3:** The comparison of relative amounts of VOCs across the samples (µg/L).

Group	Esters	Hydrocarbons	Alcohols	Ketones	Ethers	Carboxylic Acids	Heterocyclic Compounds	Others
TW	60.73 ± 5.21 ^c^	1.21 ± 0.77 ^a^	23.06 ± 0.17 ^b^	1.96 ± 0.13 ^c^	1.7 ± 0.23 ^c^	2.61 ± 3.26 ^b^	2.91 ± 0.54 ^b^	5.82 ± 1.03 ^a^
JT	55.79 ± 5.67 ^ab^	0.76 ± 0.06 ^d^	19.10 ± 0.66 ^b^	1.32 ± 0.03 ^d^	2.02 ± 0.06 ^d^	3.71 ± 2.76 ^b^	4.17 ± 0.23 ^d^	13.13 ± 1.26 ^a^
TJ	60.04 ± 2.25 ^e^	2.43 ± 2.45 ^b^	18.96 ± 2.17 ^bc^	0.88 ± 0.22 ^d^	1.11 ± 0.03 ^a^	4.83 ± 0.14 ^b^	0.83 ± 0.07 ^d^	11.00 ± 0.1 ^a^
YJ	59.93 ± 2.19 ^b^	2.72 ± 0.77 ^a^	15.65 ± 1.98 ^bc^	1.54 ± 0.06 ^c^	1.48 ± 0.25 ^b^	2.71 ± 0.61 ^c^	0.70 ± 0.09 ^e^	15.27 ± 1.99 ^c^
YOJ	49.00 ± 0.1 ^a^	1.11 ± 0.18 ^d^	26.75 ± 1.77 ^b^	1.00 ± 0.1 ^a^	2.83 ± 0.22 ^d^	5.35 ± 0.95 ^d^	1.42 ± 1.06 ^b^	12.54 ± 2.76 ^b^

Note: Different letters of a, b, c, d, and e indicate the significant differences between the samples.

## Data Availability

The original contributions presented in this study are included in the article/Supplementary Materials. Further inquiries can be directed to the corresponding author.

## Supplementary Materials

The following supporting information can be downloaded at: https://www.mdpi.com/article/10.3390/foods14101714/s1, Table S1. The Relative Odor Activity Values (ROAVs) data integration table of key differential volatile organic compounds (VOCs) in five samples. Table S2. The quantitative list of important characteristic of volatile organic compounds (VOCs) in five samples based on machine learning.
