# Supplementary material for: Machine-Learning-Assisted Aroma Profile Prediction in Five Different Quality Grades of Nongxiangxing Baijiu Fermented During Summer Using Sensory Evaluation Combined with GC×GC–TOF-MS
Source: Foods. 2025 May 12;14(10):1714. doi: 10.3390/foods14101714 (PMC12111339; doi:10.3390/foods14101714)
Supplement: Supplementary file 1 [file foods-14-01714-s001.zip › Table S1. The Relative Odor Activity Values (ROAVs) data integration table of key differential volatile organic compounds (VOCs) in five samples..pdf]

Table S1. The Relative Odor Activity Values (ROAVs) data integration table of key differential volatile organic compounds (VOCs) in five samples.

Name	CAS	Retention Index (calculated)	Lib_RI	Range of Odor Min	TW_ROAV	JT_ROAV	TJ_ROAV	YJ_ROAV	YOJ_ROAV
Benzyl alcohol	100-51-6	1888.9	1870	40927.16	0	0	0	0.00001351596	0.0000239428314
Benzeneacetic acid, ethyl ester	101-97-3	1800	1783	406.83	0	0.075406452	0	0	0.127279339
Propanoic acid, ethyl ester	105-37-3	967.0533333	953	19019.33	0.040093165	0.0665067	0.02729631	0.010675142	0.073816771
Butanoic acid, ethyl ester	105-54-4	1047.833333	1036	81.5	42.86841298	39.69975572	28.28267085	25.01686972	74.65847083
Heptanoic acid, ethyl ester	106-30-9	1345.333333	1331	13153.17	0.05028688	0.067647098	0.113485343	0.117288212	0.075536848
Octanoic acid, ethyl ester	106-32-1	1444.9	1435	12.87	50.28511019	66.26880282	85.9297745	100	53.74633906
p-Cresol	106-44-5	2095.5	2080	166.97	0.328114993	0.354439353	0.660040394	1.363870328	1.532080531
Butanoic acid	107-92-6	1643.3	1624	964.64	0.060417982	0.088448139	0.23017399	0.280277383	0.673376708
Pyrazine, 2,6-dimethyl-	108-50-9	1338.9	1328	790.79	0	0	0	0.000499451	0.00243942
Butanoic acid, 3-methyl-, ethyl ester	108-64-5	1078.333333	1068	6.89	68.56885753	68.59196371	37.62276396	22.29129379	67.7666295
Phenol	108-95-2	2018.2	2000	18909.34	0.000476099	0.000944527	0.001126083	0.001739935	0.002506638
Pyrazine, methyl-	109-08-0	1276.3	1266	121927.01	0	0	0	0	1.038316
Decanoic acid, ethyl ester	110-38-3	1647.36	1639	1122.3	0.190221963	0.03488304	0.167634941	0.362256268	0.006158332
Pentanal	110-62-3	988.3583333	979	725.41	0.010712401	0	0.011859532	0.003942073	0.029616611
Heptanal	111-71-7	1197.4	1185	409.76	0.013962741	0.014082842	0.010319599	0.006019976	0.019539928
Nonanoic acid	112-05-0	2181.8	2170	3559.23	0.001887602	0.009344924	0.005570934	0.012324787	0.018786625
Pyrazine, tetramethyl-	1124-11-4	1482.4	1469	80073.16	1.8400907	0.000044709	3.8844	0.0000508	0.000173535
2-Acetyl-5-methylfuran	1193-79-9	1606.7	1606	40870.06	0	0	0	0.000001535	0
Phenol, 4-ethyl-	123-07-9	2177.3	2187	617.68	0.009993885	0.014060074	0.016923968	0.057814982	0.052104862
Butanedioic acid, diethyl ester	123-25-1	1690	1681	353193.25	2.99522	0.00011681	0.000146173	0.000315023	0.00064776
Nonanoic acid, ethyl ester	123-29-5	1546.9	1532	3150.61	0.043761447	0.005761086	0.027687498	0.081763825	0.002164686
Hexanoic acid, ethyl ester	123-66-0	1247.72	1233	55.33	26.08320205	63.69405351	37.96430683	13.33576043	56.88703965

1-Butanol, 3-methyl-, acetate	123-92-2	1133.3	1123	93.93	5.916784973	8.86071847	4.216963017	2.263697616	10.21161328
Octanoic acid	124-07-2	2077.3	2060	2701.23	0	0	0	0.31550642	0
Octanal	124-13-0	1302.8	1289	39.64	0.001736503	0.045839209	0.052915213	0.042815446	0.084430282
Nonanal	124-19-6	1405.9	1391	122.45	0.001361261	0.033338373	0.024990215	0.038003679	0.043880249
Ethyl Acetate	141-78-6	900.3733333	888	32551.6	0.03054983	0.107264165	0.153163965	0.032863727	0.341003872
Hexanoic acid	142-62-1	1861.273333	1846	2517.16	0.343204441	0.58879764	0.419544274	0.363300237	1.219627739
Pyrazine, trimethyl-	14667-55-1	1411.8	1402	729.86	0	0.001755159	0.00157859	0.002839344	0.009902018
Benzenepropanoic acid, ethyl ester	2021-28-5	1906.92	1893	125.21	2.746153365	3.464091463	6.417018984	1.320608536	15.92501915
Phenol, 4-ethyl-2-methoxy-	2785-89-9	2050	2032	122.74	0.025166649	0	0.029451123	0.132306704	0.043158696
n-Decanoic acid	334-48-5	2268.2	2276	13736.77	0.000108499	0.000216141	0.000254026	0.001448838	0.000626215
1-Octen-3-ol	3391-86-4	1454.4	1450	6.12	0.013332648	0.727276145	0	0.304767672	3.691501375
Dimethyl trisulfide	3658-80-8	1379.15	1377	0.36	0	0	0	0.059315784	0
Pentanoic acid, ethyl ester	539-82-2	1147.7	1134	26.78	60.09153364	91.70087195	58.73893306	40.33507991	84.44981822
2-Heptanol	543-49-7	1322.2	1320	1433.94	0.016432949	0.240739659	0.162276508	0.056037449	0.430895518
Butanal, 3-methyl-	590-86-3	922.9	918	16.51	24.73493381	21.42086743	12.41075514	8.600795887	36.22445527
Phenylethyl Alcohol	60-12-8	1926.9	1907	28922.73	0.000267267	0.001091569	0.000846886	0.002251546	0.003308257
Disulfide, dimethyl	624-92-0	1080	1077	9.13	5.269141585	1.317453147	3.400900775	3.404704594	4.508992845
Hexanoic acid, propyl ester	626-77-7	1330.6	1316	12783.77	0.013010707	0.019728061	0.021515573	0.026706658	0.022652589
Hexanal	66-25-1	1092.5	1083	25.48	4.080617575	3.853742503	2.418002137	1.138064762	5.31867491
1-Propanol	71-23-8	1042.5	1036	53952.63	0.001818915	0.002636157	0.001526581	0.001023775	0.003576393
1-Butanol	71-36-3	1148.533333	1142	2733.35	0.271086446	0.415042472	0.254550841	0.161305843	0.646150139
2-Methoxy-4-vinylphenol	7786-61-0	2204.5	2188	209.3	0	0	0	0.000722328	0
Phenol, 2-methoxy-	90-05-1	1877.8	1860	13.41	0	0.006502335	0	0.012514043	0.045140283
Creosol	93-51-6	1973.1	1956	314.56	0.001994564	0	0.004765856	0.014296844	0.002512095
Propanoic acid, 2-methyl-, ethyl ester	97-62-1	974.4866667	961	57.47	10.62184485	15.24275376	6.418271753	3.390960716	15.63332144
Propanoic acid, 2-hydroxy-, ethyl ester	97-64-3	1356.05	1347	128083.8	0.000142906	0.0036803	0.002577807	0.001383322	0.008175994

Furfural	98-01-1	1479.4	1461	44029.73	0.000114693	0.004319535	0.001188781	0.004827146	0.006017243
