# Supplementary material for: Machine-Learning-Assisted Aroma Profile Prediction in Five Different Quality Grades of Nongxiangxing Baijiu Fermented During Summer Using Sensory Evaluation Combined with GC×GC–TOF-MS
Source: Foods. 2025 May 12;14(10):1714. doi: 10.3390/foods14101714 (PMC12111339; doi:10.3390/foods14101714)
Supplement: Supplementary file 1 [file foods-14-01714-s001.zip › Table S2. The quantitative list of important characteristic of volatile organic compounds (VOCs) in five samples based on machine learning..pdf]

Table S2. The quantitative list of important characteristic of volatile organic compounds (VOCs) in five samples based on machine learning.

Name	CAS	importance	Retention Index (calculated)	Lib_RI	TW	JT	TJ	YJ	YOJ
					Unit: μg/L				
3-Methyl-1-butanol	123-51-3	0.009738095	1212.66	1209	317.2829872	13.13310425	12.76724379	9.262937034	4.994820442
1,3-Dioxolane, 2-methoxymethyl-2,4,5-trimethyl-	79449-90-4	0.0068	1476.5	0	0	0	0	0	0.004803684
Ethyl 5-methylhexanoate	10236-10-9	0.006664286	1297.573333	0	5.798578019	0.291875315	0.898083197	0.955977929	0.14054483
n-Caprylic acid isobutyl ester	5461-06-3	0.0066	1562.5	1548	6.764257054	0.01538111	0.287106888	0.42136291	0
2-Propenoic acid, ethyl ester	140-88-5	0.006583333	1005	992	0.770207677	0.039139943	0.067648435	0.03017194	0.019627856
Pentanoic acid, 2-methyl-, ethyl ester	39255-32-8	0.006514286	1148.7	1141	32.75815613	1.42640656	1.347818872	0.133996735	0.282235053
Pentanoic acid, ethyl ester	539-82-2	0.006428571	1147.7	1134	955.1966829	25.35100125	28.40619729	26.95610861	14.81110146
Benzene, 1,4-dimethoxy-	150-78-7	0.005847222	1755.2	1750	0	0	0.006932611	0.013246025	0.002179432
3-Nonenoic acid, ethyl ester	91213-30-8	0.005811905	1596.9	0	2.816993191	0.013715158	0.106584778	0.424226164	0.004365503
Methyl isovalerate	556-24-1	0.005803175	1030	1019	0	0.00532519	0.003549544	0	0.001228903
Ethyl 2-hydroxy-3-phenylpropanoate	15399-05-0	0.005633333	2277.3	0	1.012088142	0.03870899	0.056932071	0.217598702	0.030489073
Nonanoic acid, ethyl ester	123-29-5	0.005388889	1546.9	1532	85.74951054	0.237566609	3.043407543	6.320048409	0.06725165
Ethyl 9-hexadecenoate	54546-22-4	0.005385714	2268.2	2283	1.831363298	0.367802864	0.729309673	0.400998295	0.054717688
2-Pentanol	6032-29-7	0.005210476	1123.1	1119	112.8836577	4.576111724	4.199140435	1.682970711	1.749108987
2-Heptanone	110-43-0	0.005	1192.3	1182	63.71682067	2.711675655	2.577509254	0.765098741	0.721900335
1-Octanol, 2-nitro-	2882-67-9	0.005	1161.5	0	0	0	0	0	0
Acetic acid, heptyl ester	112-06-1	0.00495	1386.1	1377	1.223869146	0.046395356	0.169303133	0.207595791	0.031112773
2-Propen-1-ol	107-18-6	0.004947222	1117.9	1123	0.234428136	0.014509214	0.019821464	0.040900733	0.011988725
Benzyl alcohol	100-51-6	0.004880952	1888.9	1870	0	0	0	0.013246528	0.003476763
Decanoic acid, ethyl ester	110-38-3	0.0048	1647.36	1639	122.7740672	0.550512937	6.991366737	10.09279458	0.063595317
